# JEG-3 Trophoblast Cells Influence ILC-like Transformation of NK Cells In Vitro

**DOI:** 10.3390/ijms26083687

**Published:** 2025-04-14

**Authors:** Valentina Mikhailova, Polina Grebenkina, Sergey Selkov, Dmitry Sokolov

**Affiliations:** 1 Federal State Budgetary Scientific Institution, Research Institute of Obstetrics, Gynecology and Reproductology Named After D.O. Ott, 199034 St. Petersburg, Russiaselkovsa@mail.ru (S.S.); falcojugger@yandex.ru (D.S.); 2 Department of Immunology, Federal State Budgetary Educational Institution of Higher Education, First St. Petersburg State I. Pavlov Medical University, 197022 St. Petersburg, Russia; 3 Saint-Petersburg Pasteur Institute, 197101 St. Petersburg, Russia

**Keywords:** NK cells, ILC, trophoblast, transcription factors, AhR, T-bet, Eomes, RoRα, NK-92, JEG-3

## Abstract

The uterine decidua contains NK cells differing in their characteristics from classical NK cells, as well as other populations of innate lymphoid cells (ILCs). ILC differentiation depends on the active transcription factors: ILC1 is characterized by T-bet expression, ILC2 is defined by RORα and GATA3, ILC3 expresses RORγt and AhR. We analyzed in vitro the expression of transcription factors by NK cells in the presence of trophoblast cells and cytokines and changes in NK cell cytotoxic activity. We used NK-92 and JEG-3 cell lines, which we cocultured in the presence of IFNγ, IL-10, IL-15, and TGFβ. Then, cells were treated with antibodies to AhR, Eomes, GATA-3, RORα, RORγt, and T-bet and were analyzed. We determined NK cell cytotoxicity towards K562 cells. To characterize the functional state of trophoblast cells, we estimated their secretion of TGFβ and βhCG. We showed that in the presence of trophoblasts, the expression of the classical NK cell transcription factors—Eomes, T-bet, as well as RORα, regulating ILC2 differentiation, and AhR, participating in NCR+ ILC3 formation—decreased in NK cells. RORγt expression typical for NCR- ILC3 remained unchanged. IFNγ inhibited AhR expression. IL-10 stimulated an increase in the number of T-bet+ ILC1-like cells. Both IL-10 and IFNγ suppressed RORα expression by NK cells and stimulated TGFβ secretion by trophoblasts. After coculture with trophoblast cells, NK cells reduced their cytotoxicity. These results indicated trophoblast cell influence on the acquisition of ILC1 and ILC3 characteristics by NK cells.

## 1. Introduction

Innate lymphoid cells (ILCs)’ development depends on the transcription factors active during their differentiation. Three groups are distinguished: ILC1 (which also includes NK cells), ILC2, and ILC3 [1,2]. For classical NK cells, the key transcription factors are T-bet and Eomes, with IFNγ being the main cytokine secreted [3,4]. Other ILC1s also produce IFNγ but express only T-bet [5,6,7]. ILC2 is characterized by the transcription factor GATA3, with the secretion of IL-4, IL-5, and IL-13. The third group, ILC3, includes cytotoxicity receptor-expressing ILC (Natural Cytotoxicity Receptors (NCR+)), as well as populations of NCR-ILC3 and LTi-cells. These cells commonly express the transcription factor RORγt and secrete IL-17 and IL-22 [6,7,8]. In addition to RORγt, ILC3 also expresses the transcription factor AhR [9].

NK cells are the most studied uterine ILC population. Decidual (d) NK cells differ from classical NK cells, which constitute the major pool of NK cells in peripheral blood. DNK cells exhibit reduced cytotoxic activity, increased cytokine secretion, and distinct proteomic profile and metabolic pathways [10,11,12]. During remodeling of spiral arteries, which support placental development, special intercellular interactions form in the decidual membrane between NK cells, trophoblast cells, decidual stromal cells, and decidual macrophages [13].

It remains unclear whether dNK cells play the central role in creating a unique cellular niche in the decidua that regulates trophoblast invasion, or whether trophoblast cells themselves can influence the cellular microenvironment. For example, secretory products from HTR-8/SVneo trophoblast cells produce cytokines, in particular IL-9, which stimulate the secretion of TGFβ and IL-10 by regulatory T-cells [14]. Extravillous trophoblast and cytotrophoblast cells in vitro secrete IL-2, IL-5, IL-6, IL-8, TNFα, and a soluble form of HLA-G [15]. Trophoblast cells in vitro can form organoids similar in structure to chorionic villi and are characterized by the expression of progesterone and HLA-C [16]. The binding of HLA-C and HLA-G to dNK cell receptors regulates the functional activity of the latter [17]. Therefore, contact interactions with trophoblast cells, along with cytokines secreted by them, can affect NK cell functions.

In addition to NK cells, other ILCs (ILC1, ILC2, and ILC3) are present in the uterus. ILC2 and ILC3 reside in the endometrium, with ILC1 and ILC3 in the first trimester decidua and ILC2 in decidua during the third trimester of pregnancy [2,9,17,18]. The likely function of ILC1 in the uterus is a defense against pathogens as well as IFNγ secretion [9,19]. Being abundant in the decidua at preterm and term gestation, ILC2 stimulates an inflammatory microenvironment via secreting IL-5 and IL-13 [20,21]. ILC3 in the uterus produces IL-8, IL-17, and IL-22 [19,22] and is able to induce adhesion molecule expression on decidual stromal cells thus being a regulator of leukocyte recruitment [2,19]. Thus, changes in ILC representation within the uteroplacental complex might influence reproductive function and can be associated with reproductive pathologies. For example, an increase in the number of ILC3s and especially ILC2s in the decidua correlates with a higher risk of premature birth [20,21].

ILCs exhibit some plasticity in differentiation. For example, in mice, under the influence of commensal microflora, ILC3 can express T-bet along with RORγt [23]. In mice decidua, there is a constant low expression of RORγt, with T-bet expression decreasing in early and late pregnancy, while GATA3 expression increases toward the end of pregnancy [24]. Disruption of RORγt expression in mice, however, does not affect fertility [24].

In summary, the division of ILC differentiation pathways largely depends on activated transcription factors. Different ILCs are present in the decidua. Trophoblast cells can influence the differentiation and functional activity of dNK cells. To explore the effects of trophoblast cells on the ability of NK cells to transdifferentiate into other ILCs, we analyzed the expression of transcription factors by NK-92 cells in the presence of JEG-3 trophoblast cells and cytokines.

## 2. Results

### 2.1. Transcription Factors in NK-92 Cells After Culturing with JEG-3 Trophoblast Cells

**Figure 1:**
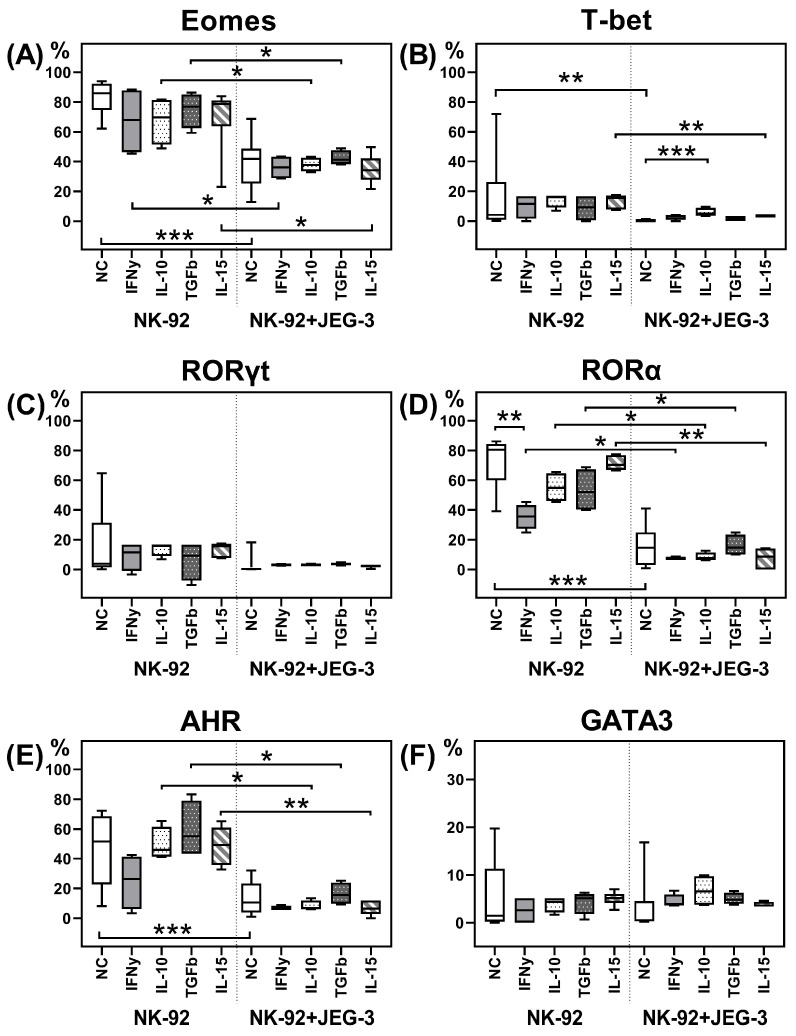
Relative number of NK-92 cells containing the transcription factors Eomes (**A**), T-bet (**B**), RORγt (**C**), RORα (**D**), AhR (**E**), and GATA3 (**F**), after cultivation without and in the presence of JEG-3 cells, without and in the presence of cytokines. NC—cultivation without cytokines. Statistical significance of differences: *—*p* < 0.05, **—*p* < 0.01, ***—*p* < 0.001.

**Figure 2:**
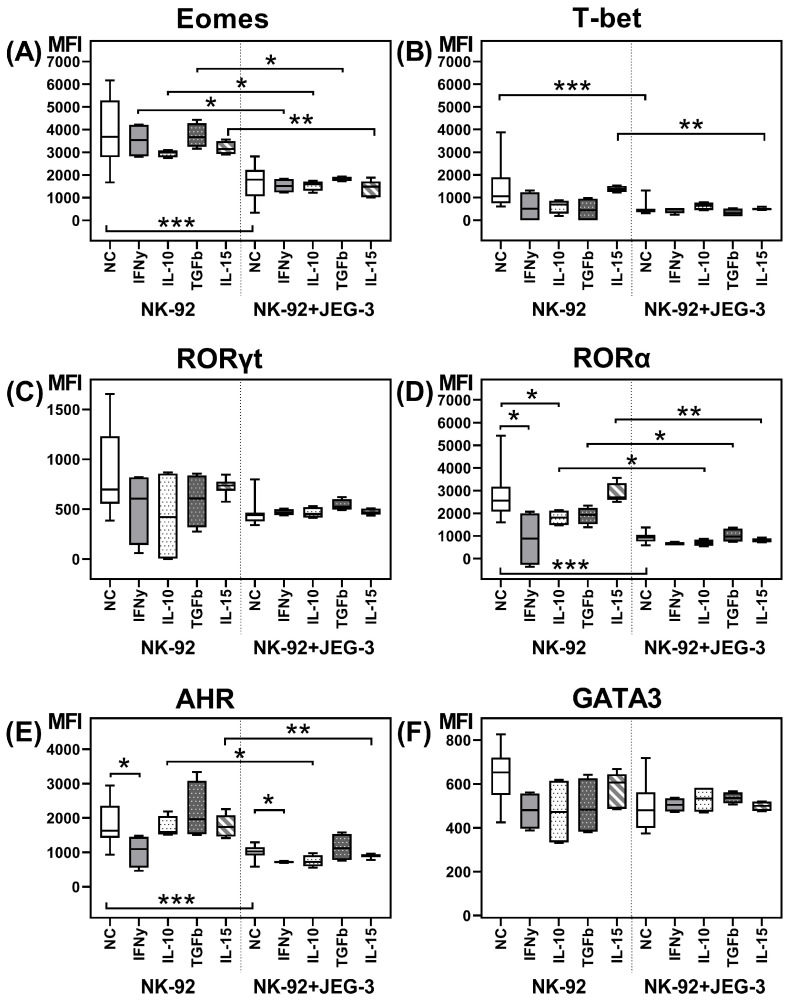
Expression intensity of the transcription factors Eomes (**A**), T-bet (**B**), RORγt (**C**), RORα (**D**), AhR (**E**), and GATA3 (**F**) by NK-92 cells after cultivation without and in the presence of JEG-3 cells, without and in the presence of cytokines. NC—cultivation without cytokines. Statistical significance of differences: *—*p* < 0.05, **—*p* < 0.01, ***—*p* < 0.001.

We estimated the relative number of NK-92 cells containing the transcription factors Eomes, T-bet, RORγt, RORα, AhR, and GATA-3, as well as the intensity of their expression after incubation in monoculture, as well as in coculture with trophoblast cells of the JEG-3 line (Figure 1A–F and Figure 2A–F). We demonstrated the expression of all the analyzed transcription factors in NK cells.

The addition of cytokines to the NK-92 cell monoculture resulted in the following changes. IFNγ reduced the relative number of RORα+ cells (Figure 1D), as well as RORα and AhR expression intensity (Figure 2D,E). IL-10 reduced only the RORα expression intensity by NK-92 cells in monoculture (Figure 2D).

After coculture with JEG-3 trophoblast cells, the relative number of NK-92 cells containing the transcription factors Eomes, T-bet, RORα, and AhR (Figure 1A,B,D,E) and their expression intensity (Figure 2A,B,D,E) reduced, compared to non-activated NK-92 cells in monoculture.

In presence of IFNγ, IL-10, TGFβ, and IL-15 in the culture medium, we showed that the inhibitory effect of trophoblast cells on the number of RORα+ and Eomes+ NK cells (Figure 1A,D) and Eomes expression intensity (Figure 2A) maintained. The reduced RORα expression intensity by NK cells in coculture was maintained in the case of IL-10, TGFβ, and IL-15 stimulation. In presence of IFNγ, we assessed no inhibitory effect of trophoblast on RORα intensity expression, probably due to its decrease in NK cells in monoculture with IFNγ (Figure 2D).

The trophoblast cell inhibitory effect on the number of T-bet+ NK-92 cells (Figure 1B) and the T-bet expression intensity (Figure 2B) was preserved only in the presence of IL-15. Cytokines IFNγ, IL-10, and TGFβ neutralized the effects of the trophoblast cells on T-bet expression by NK cells.

The inhibitory effect of trophoblast cells on the number of AhR+ NK cells preserved in coculture in the presence of IL-10, TGFβ, and IL-15 (Figure 1E). The AhR expression intensity by NK-92 cells reduced in coculture with IL-10 or IL-15 in medium. Cytokines IFNγ and TGFβ amended the suppressive effect of trophoblast cells on AhR expression by NK cells (Figure 2E).

**Figure 3:**
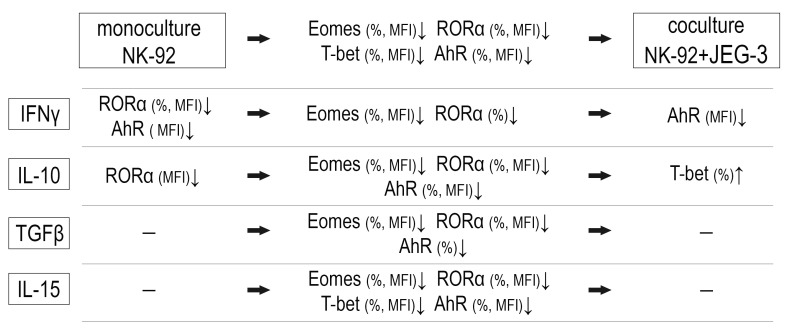
The resultant scheme of NK-92 cell expression of transcription factors in the presence of trophoblast cells and cytokines. The decrease of the transcription factor—↓, the increase of the transcription factor—↑, the coculture comparison to the monoculture—horizontal arrow.

In the coculture of NK-92 and JEG-3 cells, IL-10 stimulated an increase in the relative number of T-bet+ NK cells compared to the coculture without cytokines (Figure 1B). IFNγ suppressed the AhR expression intensity by NK cells in the coculture, compared to the coculture expression level without cytokines (Figure 2E). We summarize the obtained data on the expression of transcription factors in the scheme (Figure 3).

### 2.2. Cytotoxicity of NK-92 Cells Towards K-562 Cells After Culturing NK Cells with JEG-3 Trophoblast Cells

**Figure 4:**
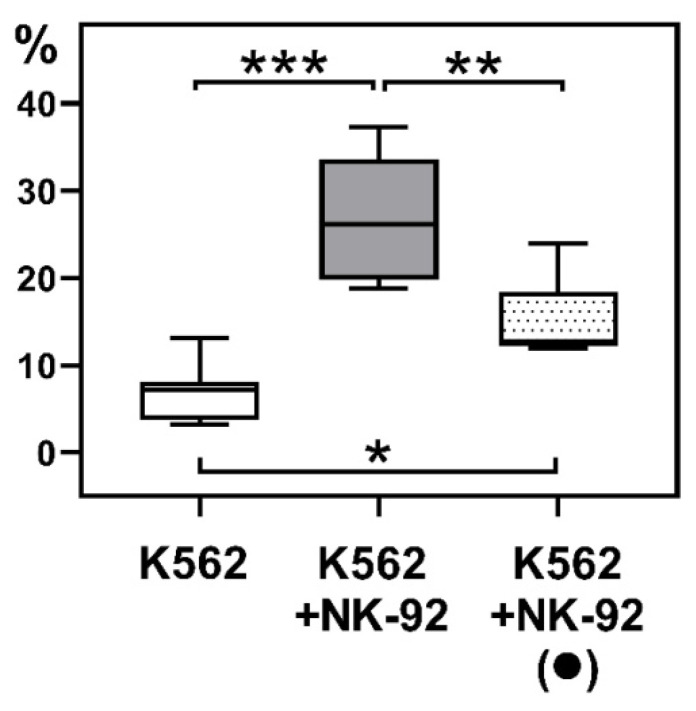
K-562 cell death after incubation with intact NK-92 cells (K562+NK-92) and NK-92 cells pre-cocultured with JEG-3 trophoblast cells (K562+NK-92 (●)). K562—baseline K562 cell death. Statistical significance of differences: *—*p* < 0.05, **—*p* < 0.01, ***—*p* < 0.001.

We showed that after preliminary culturing with JEG-3 trophoblast cells, NK-92 cells could kill K-562 target cells. However, their cytotoxic activity after coculture with trophoblast cells declined compared to intact NK cells (Figure 4).

### 2.3. Secretion of TGFβ and β-Subunit of Human Chorionic Gonadotropin (βhCG) by JEG-3 Trophoblast Cells

**Figure 5:**
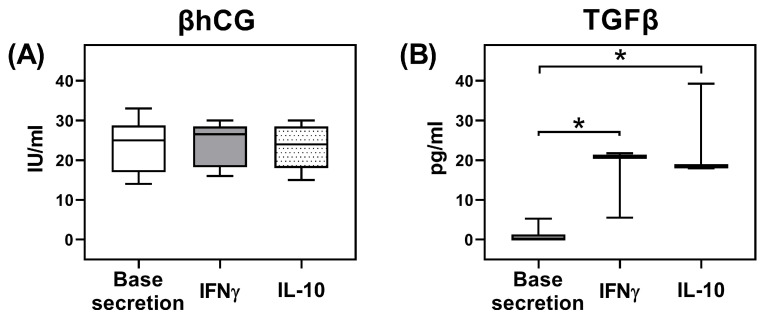
Concentration of βhCG (**A**) and TGFβ (**B**) secreted by JEG-3 trophoblast cells in the presence of IL-10 and IFNγ. Statistical significance of difference from the baseline secretion level: *—*p* < 0.05.

The secretion of βhCG by JEG-3 trophoblast cells was 25 {17; 29} IU/mL and did not change after culturing in the presence of inducers (IL-10, IFNγ) (Figure 5A). Non-activated JEG-3 trophoblast cells secreted TGFβ. After stimulation with IL-10 or IFNγ, the secretion of TGFβ by JEG-3 cells increased compared to the baseline level (Figure 5B).

## 3. Discussion

The interaction of NK cells and trophoblast cells in the uteroplacental complex is bidirectional. DNK cells regulate trophoblast cell invasion and modulate their functional characteristics [25]. In turn, trophoblast cells, through secreted chemokines, enhance the expression of adhesion receptors by NK cells and stimulate their adhesion to decidual stromal cells [26]. To investigate the potential influence of trophoblast cells on NK cell differentiation, we assessed the expression of transcription factors by NK-92 cells that are characteristic of classical NK cells and other ILC populations.

Our findings showed that in coculture with JEG-3 trophoblast cells, the number of NK-92 cells containing the transcription factors Eomes, T-bet, AhR, and RORα, as well as the intensity of their expression, decreased compared to the NK-92 cell monoculture.

These results partially align with the literature data. For example, Park S.Y. et al. previously demonstrated that trophoblast secretory factors from the Sw.71 line suppressed the production of Eomes and T-bet mRNAs in NK-92 cells [27]. The synthesis of granzyme B, perforin, and IFNγ also decreased in NK-92 cells under the influence of Sw.71 trophoblast secretory factors [27]. Additionally, other studies showed that primary trophoblast cells reduced the expression of T-bet and Helios in dNK cells, resulting in decreased IFNγ synthesis and potentially contributing to their acquisition of a regulatory phenotype [28]. Eomes and T-bet expression marks the classical NK cell population [3,4]. In this regard, the changes in Eomes and T-bet expression by NK cells that we observed after in vitro culturing with trophoblast cells can be interpreted as the inhibition of classical NK cell differentiation by trophoblasts.

According to the literature, increased AhR expression by dNK cells and peripheral blood NK cells (pNK) correlates with an increase in the cytotoxic receptors NKp30, NKp46, and NKG2D, as well as the content of GrzB and perforin [29]. CD16+ pNK cells express more AhR compared to CD16- pNK cells, and dNK cells with the CD56dim phenotype show higher AhR expression than CD56bright NK cells [29]. AhR expression is elevated in dNK cells from patients with recurrent miscarriage [29]. Thus, the observed decrease in AhR expression by NK-92 cells after coculture with trophoblast cells suggests a potential shift of NK cells towards a regulatory profile.

As previously noted, individual ILC populations are classified based on the transcription factors: T-bet expression is characteristic for ILC1, RORα and GATA-3 for ILC2, and AhR and RORγt for ILC3 [5,6,7,8]. However, a unique population of NCR- ILC3 that does not express cytotoxic receptors is formed through RORγt expression alone [6]. NCR- ILC3 appears in mucosal tissues. These cells are similar to NK cells and LTi cells in their secretion of some cytokines and receptor expression [30]. The decidua contains a subset of ILC3 that does not produce IL-22 but secretes IL-8, expresses CD127, and shares function features with dNK cells [19,30]. Decidual ILC3 (dILC3) expresses NKp44, CD161, and CXCR5 receptors more prominently than dNK cells [19]. In addition, ILC3 expresses CD117 [9].

We showed that in the presence of trophoblasts, RORα expression in NK cells decreased, suggesting inhibition of the ILC2 differentiation pathway. We also showed that RORγt expression by NK-92 cells remained unchanged in the presence of trophoblast cells. Previously, we reported an increase in the expression of CD117, CD161, and NKp44 by NK-92 cells in the presence of trophoblast cells, as well as an increase in the number of NKp44+ NK cells in peripheral blood after coculture with trophoblast cells [31]. Based on these findings and the literature data, we assume that under the influence of trophoblast cells, NK cells can acquire the characteristics of NKp44+ dILC3.

DILC3 highly expresses the transcription factor AhR [17]. We observed a decrease in AhR content in NK-92 cells after coculture with JEG-3 trophoblast cells. Thus, while RORγt expression was retained, the parallel decrease in AhR expression did not clearly indicate differentiation into ILC3, as the boundaries separating dILC3 and dNK cell populations are not well defined.

Additionally, RORγt+ ILCs can differentiate into ILC1s under the influence of IL-2 and IL-12 [32]. In the presence of mononuclear feeder cells and IL-2 or IL-15 cytokines, dILC3 shows increased proliferative activity as well as differentiation into immature NK cells [19]. Further, dILC3 differs from dNK cells in the microRNA content [33], suggesting epigenetic mechanisms underlying the formation of these cell populations. Taken together, these changes and our data on the expression of RORγt and AhR in the presence of trophoblasts suggest decidual ILC populations are in a dynamic equilibrium, influenced by the microenvironmental conditions.

The cytokine microenvironment can affect the expression of transcription factors by NK cells. Yang et al. showed that after 24 h of stimulation of dNK cells with TNFα, IL-1β, and LPS, the expression of AhR in cells increased, while IFNγ and IL-6 did not affect AhR. However, AhR mRNA expression deceased after IFNγ stimulation [29]. We found that IFNγ caused a decrease in AhR expression in NK cells, both with and without trophoblast cells. Differences in AhR expression following IFNγ exposure may be due to the exposure times to cytokine in our study and in the research by Yang et al. A longer incubation with IFNγ, carried out in our work (96 h), caused a decrease in AhR. Probably, short-term exposure to IFNγ stimulates the cytotoxic activity of NK cells, while a prolonged exposure stimulates the regulatory transformation of NK cells and downregulates AhR.

We also found that IL-10 increased the number of T-bet+ NK-92 cells in the presence of trophoblast cells. T-bet expression characterizes the ILC1 population. ILC1 shows cytotoxic activity (from weak to moderate degree), secretion of IFNγ, TNFα, IL-2, IL-4, TGFβ, and GM-CSF, and expression of cytokine receptors for IL-7, IL-17, IL-21, and TGFβ [9]. In the endometrium, ILC1 can function as a defense against pathogens [9]. IL-10 in the decidua may be produced by macrophages, dendritic cells, trophoblast cells, and dNK cells themselves [34]. Thus, our data on the increase in the number of T-bet+ NK-92 cells reflect the potential transdifferentiation of NK cells into ILC1-like cells in the presence of trophoblasts and IL-10.

The increase in ILC1 in the decidua in the first trimester of pregnancy may contribute to blastocyst invasion and placentation. In mice, some ILC populations express MHCII, such as NKp46- CCR6+ ILC3 and ILC2. The loss of MHCII expression by the NKp46- CCR6+ ILC3 population in mice is associated with infections from commensal bacteria. The defects in MHCII expression by ILC2 contribute to lower resistance to helminthic invasions [35]. Thus, the ILC1 population not only serves as a source of IFNγ that stimulates trophoblast invasion but also does not express MHCII and is incapable of presenting foreign antigens [35], which reduces the likelihood of rejection of the semi-allogeneic fetus.

ILC2, predominantly found in barrier tissues, is involved in inflammation, mucus secretion, and the disruption of barrier function after stimulation by tissue alarmins [36] and produces cytokines IL-4, IL-5, IL-10, and IL-13 [37]. ILC2s are present in the endometrium as well as in the decidua in the third trimester of pregnancy [9]; moreover, an increase in the number of ILC2, as well as ILC3 in decidua is associated with preterm births [21]. We found that in the presence of IL-10 and IFNγ, NK-92 cells reduced the expression of RORα. In addition, IFNγ caused a decrease in the number of RORα+ NK cells. Given that IL-10 and IFNγ are present in the uteroplacental complex [38,39,40], we regard the decrease in RORα expression as inhibiting ILC2 differentiation. JEG-3 trophoblast cells also inhibited RORα expression by NK cells. However, there was no cumulative effect of IL-10, IFNγ, and trophoblast cells: these cytokines did not cause a more pronounced decrease in RORα expression in NK-92 cells.

Trophoblast cells can also undergo changes as a result of interaction with NK cells. We assessed the secretion of one of the main regulatory cytokines of JEG-3 trophoblast cells—TGFβ—as well as βhCG after incubation with cytokines IL-10 and IFNγ. The selection of these cytokines for trophoblast cell stimulation is based on their secretion by both dNK cells and NK-92 cells [41,42,43]. In addition, according to the literature data, the reduced production of IL-10 and IFNγ cytokines in the decidua is associated with miscarriage [40].

Soluble factors present at the maternal–fetal interface may influence the NK cell–trophoblast system. For example, Lee C. L. et al. showed that treatment with glycodelin-A, a glycoprotein expressed in decidua, enhanced pNK cells to produce VEGF, which is characteristic for dNK cells [44]. Hu Y. et al. demonstrated that dNK cell-conditioned media enhanced HTR8/SVneo line trophoblast cells to acquire an endothelium-like morphology, produce VEGF-C, and form tube-like structures [45]. Eastabrook G. D. et al. showed that soluble factors secreted by dNK cells regulate VEGF-C production of HTR8/SVneo line trophoblast cells [46]. Ma L. et al. determined IL-8 and HGF in dNK cell conditioned media to be responsible for the induction of trophoblast invasion [47]. In the present study, we found that IL-10 and IFNγ did not change the secretion of βhCG by JEG-3 trophoblast cells, indicating the production stability of this hormone. In the presence of IL-10 and IFNγ, the concentration of TGFβ in the conditioned media of JEG-3 trophoblast cells increased. Thus, we propose that in the NK cell–trophoblast system, there may be a reciprocal cytokine influence including IL-10 and IFNγ produced by NK cells, which stimulate the TGFβ synthesis by trophoblast cells; TGFβ, in turn, can affect the functions of NK cells, for example, suppressing their cytotoxicity. Yet, further experiments using primary trophoblast cells, dNK cells, and anti-cytokine blocking antibodies are needed to support this proposal.

The literature suggests that TGFβ suppresses the transcription factor Eomes in NK cells in mice spleen and induces NK cell acquisition of an ILC1-like phenotype [48]. In humans, co-stimulation of TGFβ and IL-15 induces ILC1-like transformation of pNK cells [49]. However, according to our data, TGFβ alone did not change Eomes expression or other analyzed transcription factors by NK cells. Probably, for the modulation of NK cell differentiation, not only the effect of trophoblast secretory factors but also the contact interaction with trophoblast cells is necessary.

To determine the effects of trophoblasts on the functional activity of NK cells, we assessed the cytotoxicity of NK-92 cells after coculture with JEG-3 trophoblast cells in vitro. We demonstrated that NK cells after contact with trophoblast exhibited reduced cytotoxic activity compared to intact NK cells. Previously we assessed the cytotoxic proteins expression of NK-92 cells after culturing with JEG-3 trophoblast cells [50]. We showed the appearance of Granzyme A in JEG-3 cells after 96 h of incubation with a simultaneous decrease in its expression by NK-92 cells. Yet, the content of Granzyme B and perforin in JEG-3 cells was low following their interaction with NK cells, while the expression intensity of Granzyme A, Granzyme B, perforin, and granzyme inhibitor Serpin B9 by NK-92 decreased [50]. We assume that the observed cytotoxicity reduction can only be partially due to exhaustion after preliminary NK cell exposure to trophoblasts. We suggest that the extended coculturing with JEG-3 trophoblast cells induces the regulatory state of NK-92 cells. Our results on the modulation of NK cell activity are consistent with the literature data. Sun J. et al. demonstrated that the interaction of dNK cells with trophoblast cells via Tim-3–Galecin-9 also leads to a decrease in NK cell cytotoxicity [51]. Accordingly, trophoblast cells modulate both the expression of transcription factors by NK cells and their cytotoxic function.

In the study, we determined the modulation effect of trophoblast cells on NK cells using NK-92 and JEG-3 cell lines. We admit that possible ways of dNK cell generation are differentiation from resident hematopoietic progenitors as well as recruitment from peripheral blood [52,53]. Since the origin of the dNK cell population is a matter of debate, we consider NK-92 cells in our study as NK cells derived from peripheral blood. As for the JEG-3 cell line, despite being similar in their characteristics to other trophoblast cell lines (such as JAR, BeWo) [54] and primary extravillous trophoblast subcultures [15], JEG-3 cells can be regarded only as a model of extravillous trophoblasts. Thus, further experiments are necessary to explore whether other trophoblast cell lines affect NK cells similarly.

## 4. Materials and Methods

### 4.1. Cells

To assess changes in transcription factors characteristic for NK cells, we utilized the NK-92 cell line (ATCC, Manassas, VA, USA). The original NK-92 line was derived by Dr. Gong J.H. (Rush Cancer Institute, Chicago, IL, USA) from the peripheral blood of a patient with malignant non-Hodgkin’s lymphoma [55]. The key phenotypic and functional characteristics of these cells align with those of NK cells [54,55,56,57,58]. We cultured NK-92 cells under standard conditions recommended by ATCC in α-modified Eagle’s minimal medium (α-MEM) supplemented with 500 U/mL IL-2 (Roncoleukin, St. Petersburg, Russia).

For trophoblast cells, we used JEG-3 cells (ATCC, Manassas, VA, USA), which exhibit an epithelial morphology and possess the phenotypic and functional characteristics of invasive trophoblast in the first trimester of pregnancy [15,54,59,60,61,62,63]. This cell line was originally obtained by Dr. Peter Kohler (National Institute of Child Health and Human Development (NICHD), Rockville, MD, USA) from human choriocarcinoma cells through multiple passaging [63]. We cultured JEG-3 cells in modified Eagle’s basal medium (DMEM), following ATCC guidelines.

Additionally, we used K-562 cells (ATCC, Manassas, VA, USA), which are lymphoblasts isolated from the red bone marrow of a patient with chronic myelogenous leukemia. This cell line is commonly used as standard target cells in in vitro studies of NK cell cytotoxicity. We cultured K-562 cells in RPMI-1640 medium, according to ATCC recommendations.

Using trypan blue solution (Sigma, St. Louis, MO, USA), we assessed cell viability during cultivation and experimental procedures, ensuring it remained above 96%.

### 4.2. Inductors

The cytokines used in the work were IFNγ (1000 U/mL), IL-10 (10 ng/mL), IL-15 (10 ng/mL), and TGFβ (5 ng/mL) (R&D, Minneapolis, MN, USA). We selected the inducers in accordance with the literature [60,64,65,66,67], the recommendations of the manufacturers, and our previously obtained data using the model of contact interaction between NK cells and trophoblast cells.

### 4.3. Transcription Factors Evaluation in NK-92 Cells After Coculture with JEG-3 Trophoblast Cells

**Figure 6:**
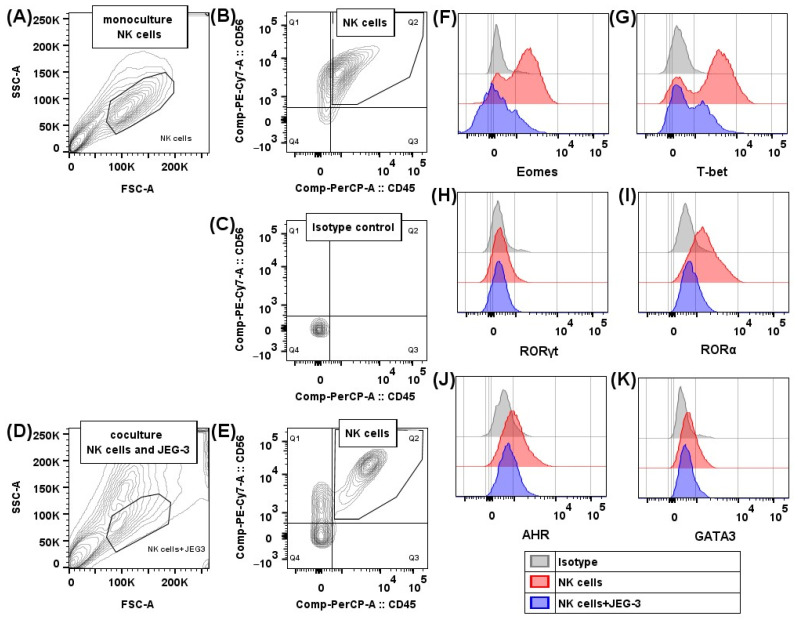
Transcription factor content in NK cells: gating strategy for NK-92 cells after cultivation without and in the presence of JEG-3 trophoblast cells. NK-92 cells in monoculture treated with antibodies to CD45 and CD56 in FSC/SSC coordinates (**A**), in CD45 PerCP/CD56 PE-Cy7 coordinates (**B**); NK-92 cells treated with isotype antibodies in PerCP/PE-Cy7 coordinates (**C**); NK-92 cells in coculture with JEG-3 trophoblast cells treated with antibodies to CD45 and CD56 in FSC/SSC coordinates (**D**), in CD45 PerCP/CD56 PE-Cy7 coordinates (**E**). Expression of transcription factors Eomes (**F**), T-bet (**G**), RORγt (**H**), RORα (**I**), AhR (**J**), and GATA3 (**K**) by NK-92 cells in monoculture (red) and coculture with trophoblast cells (blue).

We incubated JEG-3 cells in a flat-bottomed 24-well plate (Sarstedt, Numbrecht, Germany) at a concentration of 2 × 10^5^ cells per 1 mL of complete DMEM medium at 37 °C, 5% CO_2_, for 24 h until confluent monolayer formation. Then, we added NK-92 cells to the wells with a monolayer of JEG-3 cells at 2 × 10^5^ cells in 1 mL of fresh complete medium with α-MEM and IL-2 (500 U/mL), thus obtaining a coculture. We also incubated NK-92 cells in wells that did not contain JEG-3 cells, i.e., under monoculture conditions. Cytokines IFNγ, IL-10, IL-15, and TGFβ were added to some wells with mono- and coculture. After 96 h of incubation, we treated the cell suspensions with antibodies to the surface receptors CD45 and CD56 (BD, USA), following the manufacturer’s instructions. After that, we fixed and permeabilized the cells with subsequent intracellular staining using the Transcription-Factor Buffer Set reagent kit (BD, Franklin Lakes, NJ, USA) as per the manufacturer’s guidelines. The antibodies used were to the transcription factors AhR, Eomes, GATA-3, RORα, RORγt, and T-bet, with isotopic controls to monitor non-specific antibody binding. We analyzed the samples on an FACSCanto II flow cytometer (BD, Franklin Lakes, NJ, USA) (Figure 6). There were five independent experiments performed without cytokines and two experiments for each cytokine, containing two replicates.

### 4.4. Cytotoxicity Assay of NK-92 Cells After Their Coculture with JEG-3 Trophoblast Cells

We seeded JEG-3 cells to adherent cell culture flasks at a concentration of 2 × 10^5^ cells per 1 mL of medium. After 24 h, the cells were treated with 4 µmol of vital dye—carboxyfluorescein succinimidyl ester (CFSE) solution—to distinguish them later by fluorescence (Sigma, St. Louis, MO, USA). Then, we added NK-92 cells at a concentration of 2 × 10^5^ cells in 1 mL of complete α-MEM medium, IL-2 (500 U/mL). The cell culturing proceeded for 96 h to assess NK cell cytotoxic activity in parallel to transcription factors changes. Then, we separated NK-92 cells from JEG-3 cells using a FACSAria III cell sorter (BD, Franklin Lakes, NJ, USA) by the CD45+CD56+ phenotype, using the Purity protocol and a 85 μm nozzle. To reduce the negative effect of sorting on the functional activity of NK cells, we left them after sorting for 24 h in a complete α-MEM medium. After 24 h, we assessed the cytotoxicity of NK-92 cells towards target K-562 cells using the method described previously [68]. We completed four independent experiments, containing two replicates.

### 4.5. Secretion of TGFβ and β-Subunit of Human Chorionic Gonadotropin (βhCG) Evaluation by Trophoblast Cells of the JEG-3 Line

To characterize the functional state of trophoblast cells, we assessed their secretion of TGFβ and βhCG. For this purpose, we placed 20,000 cells of the JEG-3 trophoblast line into a 96-well flat-bottomed plate (Sarstedt, Austria) in 100 μL of complete DMEM medium and incubated for 24 h until a confluent monolayer formed. Then, we added the cytokines IFNγ and IL-10 to the cells and cultured them for 24 h. We chose these cytokines because they had an isolated effect on the transcription factor content in NK cells. Additionally, NK-92 cells previously demonstrated the secretion of these cytokines [42,43]. We used intact JEG-3 cells as a control. After the plates were centrifuged for 10 min at 200× *g*, we sterilely collected conditioned media from the wells and froze them at −20 °C. We used the Cytometric Bead Array method with standard CBA human TGFβ Flex Set, according to the manufacturer’s instructions (BD, Franklin Lakes, NJ, USA), to assess the content and concentration of TGFβ in the obtained samples. Before measurement, we treated the samples with a 1 N HCl solution for 10 min; then, we performed a neutralization reaction with a 1.2 N NaOH solution containing 0.5 M HEPES, according to the kit manufacturer’s recommendation (BD, Franklin Lakes, NJ, USA). We analyzed samples from three independent experiments for each cytokine inducer, containing one replicate.

We determined the βhCG content by electrochemiluminescence analysis using the commercial Elecsys βhCG test systems from Roche Diagnostics (Indianapolis, IN, USA) on a Cobas e411 automatic immunochemical analyzer (Roche Diagnostics, Indianapolis, IN, USA) in accordance with the manufacturers’ instructions. The samples from three independent experiments for each cytokine inducer, containing two replicates, were analyzed.

### 4.6. Data Statistical Processing

We performed statistical analysis in GraphPad Prism 8 (version 8.0.0 for Windows, GraphPad Software, San Diego, California, USA). The presence of abnormal values (outliers) was assessed; if any were detected, we excluded them from the analysis. We analyzed the homogeneity of variances using the Bartlett test. Since we detected their inequality, we used nonparametric statistics methods to compare the data: the Mann–Whitney rank test for pairwise comparisons and the Kruskal–Wallis test followed by Dunn’s post hoc test for multiple comparisons. We considered differences statistically significant at *p* < 0.05. We selected three options for designating the level of significance: *p* < 0.05 (*), *p* < 0.01 (**), *p* < 0.001 (***). To present the data, the values of the medians (m) and upper (75%) and lower (25%) quartiles were calculated. According to these, we constructed box diagrams, with the minimum and maximum of the distribution indicated by the “whiskers” of the box.

## 5. Conclusions

We showed that in the presence of JEG-3 trophoblast cells, the expression of transcription factors regulating the formation of classical NK cells—Eomes and T-bet—was reduced in NK-92 cells. Contact with trophoblast cells also led to a decrease in the NK cell expression of RORα, which regulates the formation of the ILC2 population, and AhR, which is involved in the differentiation of NCR+ ILC3. However, there were no changes in the expression of the transcription factor RORγt by NK-92 cells in the presence of trophoblasts. After coculture with trophoblast cells, the cytotoxic activity of NK-92 cells decreased. Cytokines affected the expression of transcription factors in NK cells. IFNγ led to a decrease in AhR expression. IL-10 stimulated an increase in the number of T-bet+ cells. Both IL-10 and IFNγ suppressed the RORα expression by NK cells but stimulated trophoblast cell secretion of TGFβ. Overall, the observed changes in the expression of Eomes, T-bet, RORα, and AhR, along with the decrease in NK cell cytotoxicity, suggests a dynamic equilibrium between NK cell and ILC populations in the decidua, changing depending on cytokine microenvironment.

## Data Availability

The raw data supporting the conclusions of this article will be made available by the authors on request.
